# Comparative evaluation of chlorous acid and sodium hypochlorite activity against SARS-CoV-2

**DOI:** 10.1099/acmi.0.000354

**Published:** 2022-05-23

**Authors:** Noritoshi Hatanaka, Sharda Prasad Awasthi, Bingting Xu, Hisataka Goda, Hiroyuki Kawata, Isanori Horiuchi, Mayo Yasugi, Shinji Yamasaki

**Affiliations:** ^1^​ Graduate School of Veterinay Science, Osaka Metropolitan University, 1-58, Rinkuourai-kita, Izumisano, Osaka 598-8531, Japan; ^2^​ Asian Health Science Research Institute, Osaka Metropolitan University, 1-58, Rinkuourai-kita, Izumisano, Osaka 598-8531, Japan; ^3^​ Osaka International Research Center for Infectious Diseases, Osaka Metropolitan University, 1-58, Rinkuourai-kita, Izumisano, Osaka 598-8531, Japan; ^4^​ Sankei Co. Ltd., 2-2-53, Siromi, Chuou-ku, Osaka 540-0001, Japan

**Keywords:** Chlorous acid, Sodium hypochlorite, SARS-CoV-2

## Abstract

A novel coronavirus, named severe acute respiratory syndrome coronavirus 2 (SARS-CoV-2), suddenly emerged in China in 2019, spread globally and caused the present COVID-19 pandemic. Therefore, to mitigate SARS-CoV-2 infection effective measures are essential. Chlorous acid (HClO_2_) has been shown to be an effective antimicrobial agent. However, at present there is no experimental evidence showing that HClO_2_ can inactivate SARS-CoV-2. Therefore, in this study, we examined the potential of HClO_2_ to inactivate SARS-CoV-2 in presence or absence of organic matter and the results were compared with that of sodium hypochlorite (NaClO), another potent antimicrobial agent. When concentrated SARS-CoV-2 was incubated with 10 ppm HClO_2_ for 10 s, viral titre was decreased by 5 log of 50% tissue culture infective dose per mL (TCID_50_ ml^−1^). However, the same concentration of NaClO could not inactivate SARS-CoV-2 as effectively as HClO_2_ did even after incubation for 3 min. Furthermore, 10 ppm HClO_2_ also inactivated more than 4.0 log of TCID_50_ within 10 s in the presence of 5 % fetal bovine serum used as mixed organic matters. Our results obtained with HClO_2_ are more effective against SARS-CoV-2 as compared to NaClO that can be used for disinfectant against SARS-CoV-2 .

## Introduction

A novel coronavirus was identified in patients with pneumonia in China at the end of 2019 and named as severe acute respiratory syndrome coronavirus 2 (SARS-CoV-2) [1, 2]. This virus has quickly spread all over the world and the disease caused by SARS-CoV-2 is termed coronavirus disease 2019 (COVID-19). World Health Organization (WHO) announced COVID-19 as a pandemic on March 2020 [3]. As of 23 November 2021, more than 257 million cases and 5.1 million deaths due to SARS-CoV-2 infection have been confirmed globally [4].

SARS-CoV-2 infection is considered to be transmitted from human to human through droplets and close contact, and is most probably an airborne infection [5]. Therefore, inactivation of the virus in droplets appears to be important in preventing the spread of the disease since SARS-CoV-2 could survive more than 5 days on plastic and stainless steel with infectivity [6]. Although vaccination for COVID-19 has contributed to the reduction of number of infected persons and death in the countries such as USA, Israel and UK, where more than 60% of the people were vaccinated, there are still a number of newly infected persons reported every day, especially where the vaccination rate is less. In addition, novel variants such as alpha, beta, gamma, delta and very recently omicron have emerged and spread globally [7].

To avoid the transmission of SARS-CoV-2, WHO has recommended 1000 ppm sodium hypochlorite (NaClO), which has commonly been used as a disinfectant on surfaces which might be contaminated with SARS-CoV-2 [8]. However, 1000 ppm NaClO may cause metal corrosion, irritation of mucous membranes, etc. [9]. Furthermore, it has been reported that the antimicrobial activity of NaClO decreases in the presence of organic matter [10].

Chlorous acid (HClO_2_) is also a common disinfectant and it has been recognized as an ethical pharmaceutical for sanitizing. Several studies have showed that HClO_2_ has potent bactericidal activity against pathogenic organisms including *Staphylococcus aureus *, *Escherichia coli *, *Candida albicans*, etc. [11]. Furthermore, Goda *et al.* [12] have reported that HClO_2_ can inactivate enveloped viruses, such as influenza virus A and herpes simplex virus type 1. In fact, HClO_2_ exhibits much stronger antiviral activity against enveloped viruses compared to that of non-enveloped viruses [12]. However, at present it is not known whether HClO_2_ can efficiently inactivate SARS-CoV-2. Even if it does, there is no information about how much and for how long HClO_2_ is needed to inactivate SARS-CoV-2. Therefore, in this study, we have experimentally tested the potential of HClO_2_ to inactivate SARS-CoV-2 in the presence or absence of organic matter, and the results were compared with that of NaClO.

## Methods

VeroE6/TMPRSS2 cells [13], purchased from Japanese Collection of Research Bioresources (Osaka, Japan), were used for cultivation of SARS-CoV-2. VeroE6/TMPRSS2 cells were cultured at 37 °C in 5% CO_2_ in air in Dulbecco’s modified eagle medium, low glucose, pyruvate (DMEM; Thermo Fisher Scientific Inc., Waltham, MA, USA) supplemented with 5 % heat inactivated fetal bovine serum (FBS；Thermo Fisher Scientific Inc.) and 1 mg ml^−1^ G418, which is geneticin commonly used as a selective agent for eukaryotic cells (Nacalai Tesque, Inc., Kyoto, Japan).

One hundred and forty thousand of VeroE6/TMPRSS2 cells were cultured in a 25 cm^2^-cell culture flask at 37 °C for 16 h in a 5 % CO_2_ humidified incubator. Cells were infected with MOI=0.001 of SARS-CoV-2 JPN/TY/WK-521 strain and incubated at 37 °C for 48 to 96 h in DMEM (Thermo Fisher Scientific Inc.,) supplemented with 2 % heat inactivated FBS (Thermo Fisher Scientific Inc.) and 1 mg ml^−1^ G418 (Nacalai Tesque, Inc.). After cytopathic effect (CPE) was observed, the spent culture medium was harvested and centrifuged at 3000 r.p.m. for 5 min (LC-220; Tomy Seiko Co., LTD., Tokyo, Japan) followed by collection of the supernatant fraction containing virus particles. Then, 1 g of polyethylene glycol 6000 and 233 mg of NaCl (Nacalai Tesque, Inc.) were added to 10 ml of collected virus solution and kept at 4 °C for 16 h. After that, the virus solution was centrifuged at 15000 r.p.m. at 4 °C for 10 min (MX-301; Tomy Seiko Co., LTD.), supernatant was discarded and pellet was suspended in 1 ml of PBS (-) at pH 7.4. Experiments with live SARS-CoV-2 viruses were carried out at Bio-safety Level three laboratory at Osaka Prefecture University after obtaining the permission from the Biological Safety Committee of Osaka Prefecture University.

Sixty microlitres of concentrated virus solution or 30 µl of concentrated virus and 30 µl of 0 or 10% FBS in PBS (-) at pH 7.4 was mixed with 540 µl of several concentrations (10, 5 or one ppm) of HClO_2_ (Chlorous acid N barrier, Sankei Co., Ltd., Osaka, Japan) or NaClO (Wako Pure Chemical Industries, Ltd., Osaka, Japan). Then, samples were incubated at 25 °C for 10 s, 30 s, 1 min or 3 min. After incubation, 30 µl of 0.1 M sodium thiosulfate, 60 µl of 10 x DMEM (Nissui Pharmaceutical Co., Ltd., Tokyo, Japan), 12 µl of FBS and 12 µl of 50 mg ml^−1^ G418 disulfate aqueous solution were immediately added. Then, ten-fold dilution was carried out with DMEM supplemented with 2% FBS and 1 mg ml^−1^ G418, and titration was done as described below.

About 2.5×10^4^ cells 100 µl^−1^ of VeroE6/TMPRSS2 cells were seeded in a 96 well plate and cultured at 37 °C for 16 h in their respective medium. Culture medium was removed and 100 µl of 10-fold serially diluted virus in DMEM supplemented with 2 % FBS and 1 mg ml^−1^ G 418 was added. The infected VeroE6/TMPRSS2 cells were cultured at 37 °C for 72 h. Cells were fixed with methanol (Nacalai Tesque, Inc.), stained with 0.5 % crystal violet stain (Nacalai Tesque, Inc.). Then, 50% tissue culture infective dose (TCID_50_) was calculated by employing the Behrens-Kaerber method [14].

Statistical analyses were carried out using Microsoft Excel 2019 (Microsoft, Redmond, WA, USA). Error bars denote standard deviations. *p* value was determined with Student’s *t*-test using paired, two-tailed distribution. Results were considered statistically significant when *p* value showed less than 0.05 in difference of data.

## Results and discussion

SARS-CoV-2 virus has quickly spread all over the world soon after the first patient was identified in Wuhan, China in 2019. Although several types of COVID-19 vaccines have been developed and the number of infected persons drastically decreased where COVID-19 vaccines were introduced, new variants, however, emerged in various countries such in United Kingdom (alpha), South Africa (beta), Brazil (gamma) and India (delta) [7] and it is not yet clear how vaccination can contribute to convergence of COVID-19. WHO has recommended 1000 ppm NaClO or 70% ethanol for disinfecting SARS-CoV-2 [8]. However, 70% ethanol may cause skin irritation in some individuals and 1000 ppm NaClO cannot be used for humans. Therefore, it is important to provide alternative measures which can be used for human to help mitigating the COVID-19 pandemic.

**Fig. 1.**
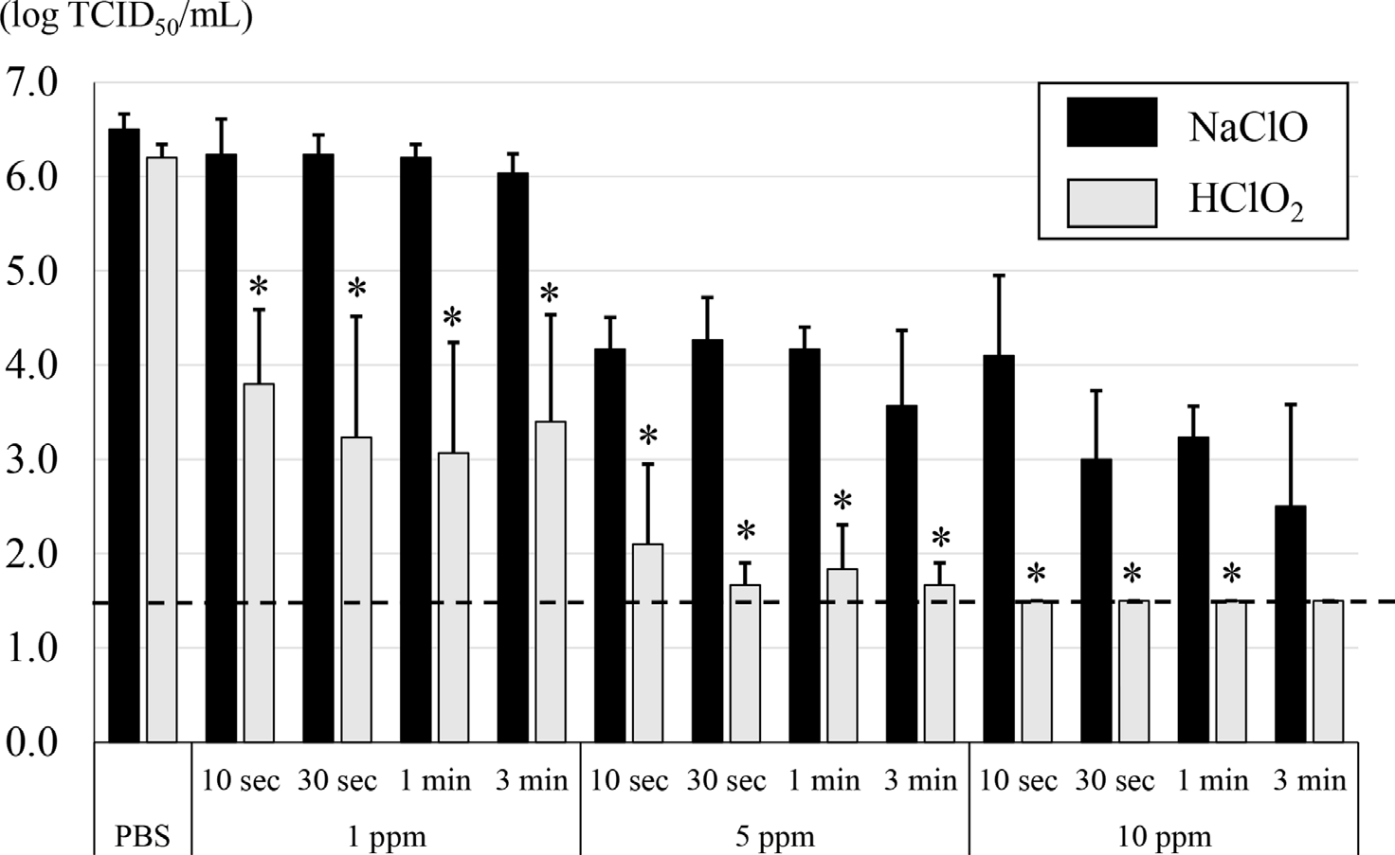
Antiviral activity of 10, 5 or 1 ppm HClO_2_ and NaClO, respectively, against SARS-CoV-2. Concentrated SARS-CoV-2 viruses were treated with 10, 5 or 1 ppm HClO_2_ and NaClO for 10 or 30 s and 1 or 3 min, respectively, followed by determination of viral titre as 50 % tissue culture infective dose per millilitre (TCID_50_ ml^−1^). Dotted line indicates detection limit (≦1.5 log TCID_50_ ml^−1^). All data represent the means±standard deviation from three independent experiments. ^*^Viral titre is significantly different between HClO_2_ and NaClO. No toxicity was observed when 10 ppm of each chemical was applied to VeroE6/TMPRSS2 cells.

**Fig. 2.**
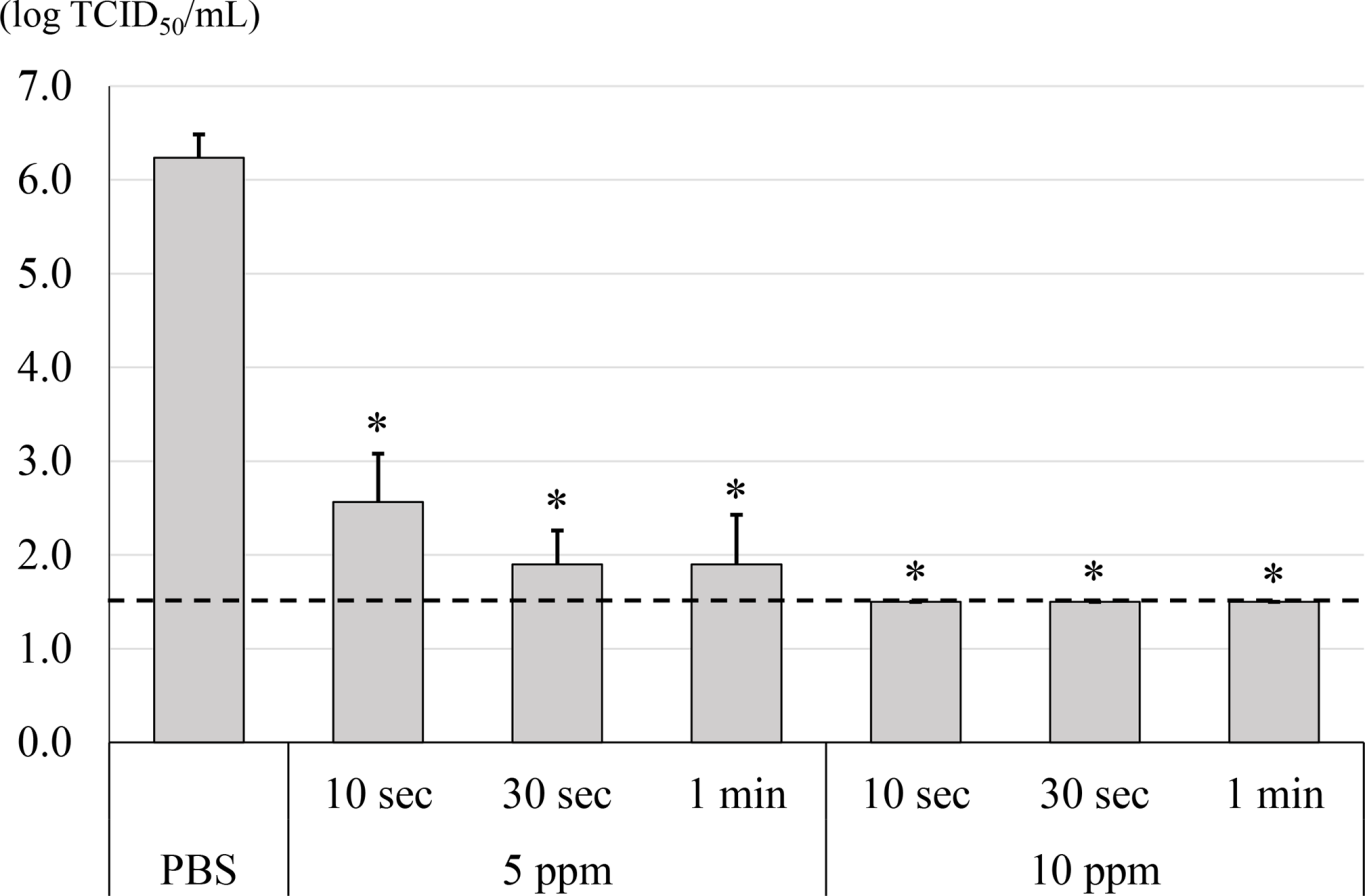
Antiviral activity of 10 or 5 ppm HClO_2_ against SARS-CoV-2 in the presence of 5 % FBS in virus solution. Concentrated SARS-CoV-2 with 5% FBS was treated with 10 or 5 ppm HClO_2_ for 10, 30 s or 1 min followed by determination of viral titre with 50% tissue culture infective dose per millilitre (TCID_50_ ml^−1^). Dotted line indicates detection limit (≦1.5 log TCID_50_ ml^−1^). All data represent the means±standard deviation from three independent experiments. ^*^Viral titre is significantly different between HClO_2_ and PBS. No toxicity was observed when 10 ppm of each chemical was applied to VeroE6/TMPRSS2 cells.

HClO_2_ is one of the most common chlorine-based disinfectants and can be used for humans. However, there is no direct evidence showing that HClO_2_ can inactivate SARS-CoV-2. Therefore, in this study, HClO_2_ was extensively evaluated to assess its potential to inactivate SARS-CoV-2 compared to that of NaClO. When SARS-CoV-2 viruses were treated with HClO_2_, viral titre was decreased in a dose dependent manner (Fig. 1). For example, it was observed that 1 ppm HClO_2_ reduced viral titre by 2 log TCID_50_ ml^−1^ within 10 s. When incubation continued for more than 30 s, viral titre decreased by about 3 log TCID_50_ ml^−1^. However, 5 ppm HClO_2_ reduced viral titre by 4 log TCID_50_ ml^−1^ within 10 s. When incubation continued for more than 30 s, viral titre decreased by about 4 log TCID_50_ ml^−1^ or more in the absence as well as presence of 5% FBS (0.5 % as a final concentration). Furthermore, 10 ppm HClO_2_ reduced viral titre to the level of detection limit (≦1.5 log TCID_50_ ml^−1^) within 10 s regardless of presence or absence of 5% FBS (0.5% as a final concentration) (Figs. 1 and 2). Since protein concentration of saliva is known to be around 1.1 mg ml^−1^ [15], 5% FBS contains two times higher protein concentration than that in saliva. However, when viruses were treated with 1 ppm NaClO the viral titre was not decreased even after prolonged incubation for 3 min. On the other hand, viral titre was decreased by 3 and 4 log TCID_50_ ml^−1^ when the viruses were treated with 5 and 10 ppm NaClO for 3 min, respectively (Fig. 1). It should be noted that the use of highest concentration of HClO_2_ and NaClO, i.e. 10 ppm, did not show any cytotoxicity under the experimental conditions. Altogether, these data suggest that HClO_2_ is a more potent antiviral reagent against SARS-CoV-2 than NaClO.

Goda *et al.* [12] have reported that HClO_2_ inactivated both influenza virus A and herpes simplex virus type 1, indicating that HClO_2_ is not only specifically effective to SARS-CoV-2 but it also works against other viruses. In our previous study, we have examined the antibacterial activity of HClO_2_ against *Campylobacter jejuni* and *C. coli *, and compared with that of NaClO. The data also indicated that HClO_2_ has more potent antibacterial activity than NaClO regardless of presence and absence of organic matter [16]. Although the concentration of disinfectants used in this study was very low in comparison to that in the protocol of WHO [8], it is important to know the minimum antiviral concentration of disinfectant because an overdose of any disinfectant may be harmful for humans as well as for the environment.

## Conclusion

Our data revealed that HClO_2_ can inactivate SARS-CoV-2 in lower concentration in comparison to NaClO and may be a more potent disinfectant against SARS-CoV-2 than NaClO regardless of presence and absence of organic matter although infection dose of SARS-CoV-2 to human is unknown. Effectiveness of low concentration of HClO_2_ against SARS-CoV-2 is a big practical advantage. Further studies are going on in our laboratory to know the efficacy of HClO_2_ against SARS-CoV-2 variants in presence or absence of organic matters such as saliva.
